# Notes and News

**Published:** 1903-04-04

**Authors:** 


### Notes and News.

The King and Queen have intimated that they will be happy to attend the afternoon service at St. Paul's Cathedral on Sunday, June 7th, on which day the collections at the services in the Cathedral will be devoted to the hospitals and dispensaries of the metropolis through the Hospital Sunday Fund. The Lord Mayor and the Cathedral authorities will make the necessary preparations for their Majesties' visit.

The Queen has sent a special donation of ten guineas to the Hospital and Home for Incurable Children, Maida Vale, towards the special building fund.

The Prince of Wales has selected Tuesday, May 19th, 1903, to open the Passmore-Edwards Sailors' Palace, the new headquarters of the British and Foreign Sailors' Society, situated in East London. The Prince will be accompanied by the Princess of Wales. Mr. Passmore Edwards, who has given £14,000 towards Jack's palace, will also found the Ocean Library connected with it by presenting a nucleus of 2,000 volumes.

Messrs. Bovril are presenting a fine engraving of Lord Kitchener's Home Coming, from a painting by Mr. W. Hatherall, R.I., in exchange for coupons distributed with Bovril, the offer being open until June 30th. Purchasers of a little over four pounds of Bovril can possess one of the pictures.

The report of Dr. Barclay Dick, medical officer for a gold mine in Ashanti, illustrates what can be done under intelligent direction when an English settlement is formed in a malarious country. Under Dr. Dick's direction a model township was established, and all the workers at the mines removed to it, with the result that malarial fever has been practically banished amongst the inhabitants.

The late Mr. Henry Baker Hicks has bequeathed £1,000 each to the Bristol Royal Infirmary and the Bristol General Hospital, and smaller sums to other British charities.

The Academy of Medicine of Paris issues a warning against apéritifs as a dangerous form of alcohol consumption, as they are taken fasting. The use of these beverages is not so popular in this country as in France. The American cocktail, of course, is equally dangerous, and an "Anti-drink Between Meals Society" could carry on a very active crusade, not only in France and America, but also in Australia and India, where the habit of constant drinks is firmly rooted.

On Friday, February 13th, the new Hospital and Dispensary for Women, Wuchang, Hankow, China, was opened by the Rev. Arnold Foster, M.A. Over seventy guests came to show their appreciation of the work. Among those present were Dr. Martin (author of "The Lore of Cathay"), Dr. and Mrs. Davenport, Bishop and Mrs. Ingle, Dr. and Mrs. Sydney Hodge, Rev. and Mrs. S. S. Warren, Dr. and Mrs. Huntly, Dr. and Mrs. Boothe, Dr. and Mrs. Gilleson, Mrs. Craddock, Miss Calvert, Miss Margaret Bennett, L.S.A., medical superintendent; and Miss Jean S. Shillington, matron. The hospital fittings and buildings are the property of the Wesleyan Missionary Society.

The London County Council, at the recommendation of their Public Health Committee, have made an order applying for the inclusion of measles in their regulations relating to dangerous infectious diseases, to come into effect on and after April 1st. Under the order full power is given to sanitary authorities to enforce cleansing and disinfection of houses and their contents, and to impose a fine for non-compliance. The order prohibits the letting of infected premises, the exposure of infected persons, forbids such persons from carrying on any occupation likely to spread disease; the conveyance of infected persons in public carriages; and the retention of the body of a person who has died of an infectious disease within a dwelling house for more than 48 hours. Penalties are attached to all infringements of these and other regulations we have not space to enumerate.

The Semi-Teetotal Pledge Association, the President of which is Lord Roberts, has sent for our inspection the official button intended to be worn by its followers. The button is in the form of a large stud of metal enamelled, upon which is inscribed in red letters "No Drinks between Meals." We are glad to learn that the Association has already a following of 17,000, but we are not so sure if the whole of the 17,000 parade the trade mark of virtue by wearing a conspicuous badge, that courage will be imparted to the weaker brethren by so doing; possibly that may serve as a reason, but we doubt its efficacy. It will probably provoke that ridicule and banter which the weak cannot stand. After all, the movement is purely a matter of common sense and hygiene, and it will be a pity to endanger its principles by associating with fads and intolerance, an association which has always stood in the way of teetotalism.

